# Genetic and Pharmacological Targeting of CSF-1/CSF-1R Inhibits Tumor-Associated Macrophages and Impairs BRAF-Induced Thyroid Cancer Progression

**DOI:** 10.1371/journal.pone.0054302

**Published:** 2013-01-23

**Authors:** Mabel Ryder, Matti Gild, Tobias M. Hohl, Eric Pamer, Jeff Knauf, Ronald Ghossein, Johanna A. Joyce, James A. Fagin

**Affiliations:** 1 Human Oncology and Pathogenesis Program, Memorial-Sloan-Kettering Cancer Center, New York, New York, United States of America; 2 Department of Medicine, Memorial-Sloan-Kettering Cancer Center, New York, New York, United States of America; 3 Department of Infectious Diseases Service & Immunology Program, Memorial-Sloan-Kettering Cancer Center, New York, New York, United States of America; 4 Department of Pathology, Memorial-Sloan-Kettering Cancer Center, New York, New York, United States of America; 5 Department of Cancer Biology & Genetics, Memorial-Sloan-Kettering Cancer Center, New York, New York, United States of America; Cardiff University, United Kingdom

## Abstract

Advanced human thyroid cancers are densely infiltrated with tumor-associated macrophages (TAMs) and this correlates with a poor prognosis. We used *BRAF*-induced papillary thyroid cancer (PTC) mouse models to examine the role of TAMs in PTC progression. Following conditional activation of *BRAF^V600E^* in murine thyroids there is an increased expression of the TAM chemoattractants *Csf-1* and *Ccl-2*. This is followed by the development of PTCs that are densely infiltrated with TAMs that express *Csf-1r* and *Ccr2*. Targeting CCR2-expressing cells during BRAF-induction reduced TAM density and impaired PTC development. This strategy also induced smaller tumors, decreased proliferation and restored a thyroid follicular architecture in established PTCs. In PTCs from mice that lacked CSF-1 or that received a c-FMS/CSF-1R kinase inhibitor, TAM recruitment and PTC progression was impaired, recapitulating the effects of targeting CCR2-expressing cells. Our data demonstrate that TAMs are pro-tumorigenic in advanced PTCs and that they can be targeted pharmacologically, which may be potentially useful for patients with advanced thyroid cancers.

## Introduction

Tumor-associated macrophages (TAMs) have the capacity to impede (M1 type) or promote (M2 type) tumorigenesis [1]. The observation that an increased density of TAMs correlates with poor clinical outcome in the majority of human cancers suggests TAMs are pro-tumorigenic [2]–[5]. Moreover, gene signatures from the stroma of human breast cancers derived from patients with poor outcome are independent prognostic markers and contain several highly expressed TAM-related genes [6]. Direct evidence of the role of TAMs in tumorigenesis is observed in breast cancer mouse models in which colony-stimulating factor-1 (CSF-1)-dependent TAM recruitment is required for tumor initiation through the stimulation of tumor angiogenesis [7]. At metastatic sites, CCR2^+^ inflammatory macrophages (Mφ) facilitate breast cancer cell extravasation, seeding and growth [8].

Multi-stage murine models of tumorigenesis are ideally suited to investigate the functional role of stromal cells on tumor initiation and progression. The best characterized of these are the RIP1-Tag islet cell carcinoma [9] and the polyoma middle T antigen (PyMT) breast cancer models [10]. These have provided valuable information, although arguably they are not genetically accurate recapitulations of the drivers of tumorigenesis in these cell types. Oncogenic mutations of *BRAF* are the most common genetic events in melanomas [11] and papillary thyroid cancers (PTC) [12], [13], and are thought to be involved in early stages of tumor development, as they are present in benign nevi [14] and micropapillary thyroid cancers [15], respectively. *BRAF* mutations are also highly prevalent in advanced forms of thyroid cancer [16], and are associated with greater mortality [17]. Interestingly, an increased density of TAMs correlates with lymph node metastases in PTCs [18] and invasive disease and decreased survival in poorly differentiated thyroid cancers (PDTC) [4]. Indeed, TAMs comprise at least 50% of the tumor volume of most anaplastic thyroid cancers (ATC), an extremely virulent form of the disease that is almost invariably fatal [4], [19], [20].

Several mouse genetic models of *BRAF*-induced cancer have been developed, either by activation of latent endogenous mutant alleles [21], [22] or through thyroid-specific overexpression [23]. All show high penetrance of thyroid cancer, and progression to poorly differentiated tumors. Moreover, conditional activation of BRAF in murine thyroids induces PTC that is completely reversed upon de-induction of BRAF [24].

In this study, we show that activation of oncogenic BRAF in murine thyroids results in an increased expression of the chemoattractants CSF-1 and CCL2 and that this is associated with a rapid and robust recruitment of TAMs that express the cognate receptors *Csf-1r* and *Ccr2*, respectively. Selectively targeting CSF-1 or CCR2-expressing cells during BRAF activation significantly reduced TAM density and impaired PTC development and progression. The depletion of TAMs during early PTC development impaired stromal expansion of cancer-associated myofibroblasts (CAFs). In established PTCs, tumors develop tall cell and poorly-differentiated foci, which are histological features that are characteristic of advanced human thyroid cancers. Targeting CCR2-expressing cells in mice with advanced PTCs resulted in a reduced TAM density as well as smaller, more differentiated thyroid cancers with fewer foci of tall cells and poorly-differentiated areas. Pharmacological targeting of BRAF-induced PTCs with a selective c-FMS/CSF-1R kinase inhibitor recapitulated the effects of targeting CCR2-expressing cells. These data suggest that therapeutic strategies targeting TAMs may be beneficial in this disease, and perhaps enhance the efficacy of kinase inhibitors directed against the cell autonomous oncogenic drivers of the disease.

## Materials and Methods

### Mouse models

The following two models of *BRAF*-induced thyroid cancer were used in this study: *1) Tg-rtTA/tetO-BRAF^V600E^* mice express oncogenic BRAF^V600E^ in thyroid cells in a dox-dependent manner, and were maintained in an FVB/N background [24]. 2) *Tg-Braf* transgenic mice express the human oncoprotein under the control of the bovine thyroglobulin gene promoter [23]. *Ccr2-DTR* and *Ccr2-GFP* mice express the DTR or green fluorescent protein (GFP), respectively, under the control of the monocyte/Mø-specific *Ccr2* gene promoter, and were maintained in a C57/B6 background [25]. *Csf-1^−/−^* mice (Jackson Lab, Bar Harbor, ME) are deficient in circulating and tissue monocytes/Mφ [26], [27]. All animal husbandry and experimental procedures were approved by the Memorial-Sloan Kettering Cancer Center Institutional Animal Care and Use Committee.

### Depletion of TAMs in Braf-induced thyroid cancer mouse models

Macrophage depletion in the bone marrow (BM), blood, spleen and peritoneal cavity was examined in *Ccr2-DTR* mice after treatment with diphtheria toxin (DT) 20 ng/g (List Biologicals, Campbell, CA ) intraperitoneally (i.p.) on alternating days for 7 days. Twenty-four hours following the last dose of DT, mice were euthanized by CO_2_ inhalation and tissue samples obtained for flow cytometry and/or immunohistochemistry (IHC) as described below.

To assess the effects of TAMs on PTC development, *Tg-rtTA/tetO-Braf/Ccr2-DTR* mice were fed dox-impregnated chow (2500 ppm; Harlan-Teklad) for 7 days with or without DT i.p. on alternating days beginning on day 0. On day 7 (24 hr after the last dose of DT), mice were euthanized by CO_2_ inhalation and thyroids extracted for IHC. To examine the role of TAMs in established BRAF-induced thyroid cancers we treated *Tg-Braf/Ccr2-DTR* mice at approximately 6 and 12 weeks of age with or without DT on alternating days for 10 days. Mice were euthanized and thyroids extracted 24 hr after the last dose of DT for flow cytometry and IHC.

### FACS Analysis

Pooled thyroids were harvested after intracardiac PBS perfusion to deplete circulating leukocytes. Thyroids were minced into small fragments followed by enzymatic digestion into single cell suspensions with collagenase type 2 (Worthington, Lakewood, NJ) and dispase (Invitrogen, Carlsbad, CA) for 90 minutes in a shaking incubator at 37°C. Samples were then washed three times with ice-cold media supplemented with 10% FBS followed by reconstitution in FACS buffer (PSB/1% BSA). Samples were blocked with mouse Seroblock FcR (AbD Serotec, Raleigh, NC) for 10 minutes on ice followed by a 30 minute incubation with the following fluorescently conjugated antibodies: Cd45:PerCP Cy5.5, Cd11b:APC, Gr-1:FITC, Ly6C:FITC, Ly6G:FITC (BD Pharmingen, San Diego, CA ) & F4/80:FITC (AbD Serotec). Single cell suspensions of BM aspirates, blood samples that were cleared of red blood cells and peritoneal lavages were blocked and labeled as above. Data collection was obtained using the FACS Caliber flow cytometer through the MKSCC Flow Core and data analysis was performed using Flowjo 7.2.5 software.

TAMs from thyroid cancers of *TPO-Cre/LSL-Braf* mice, which express endogenous levels of BRAF^V600E^ [21], were sorted with Cd11b:APC from thyroid single cell preparations as described above.

### Immunofluorescence/Immunohistochemistry (IHC)

For immunofluorescence serial sections were obtained from fresh frozen, OCT-embedded thyroids and/or spleens. Thyroid 5 µM sections from at least 3–4 levels, each ∼150 µm apart, were obtained. Slides were air dried, fixed with ice cold acetone for 30 minutes, re-dried and then placed in PBS. Sections were blocked with DakoCytomation serum-free protein block (Dako, Carpinteria, CA) for 30 minutes followed by PNB blocking reagent (Perkin Elmer, Waltham, MA) for 60 minutes. The following mouse primary, unconjugated antibodies were used: Cd11b, Cd68 and αSMA followed by incubation with secondary antibodies of either Alexa-Fluor 488 or Alexa-564. Sections were imaged on an upright Zeiss Axio2Imaging microscope at the MSKCC Molecular Cytology Core Facility.

For IHC, thyroids were fixed overnight at 4°C with fresh 4% paraformaldehyde using continual rotation. Tissues were washed with 2 cycles of 30 min PBS incubations and then placed in 70% ethanol. Tissues were processed into paraffin-embedded blocks followed by serial sectioning. After deparaffinization, H&E and immunostains were performed either manually (Laboratory of Comparative Pathology) or automated (Molecular Cytology Core Facility) using a Discovery XT processor (Ventana Medical Systems). The following antibodies were used: Ki67 (VP-K451, Vector Laboratories), pERK (#9101, Cell Signaling Technology), Mac-2 (#CL8942B, Cedarlane), alpha smooth muscle actin (αSMA) (# M0851, Dako) and Iba-1 (cat# 019-19741, Wako). TUNEL assay was performed as previously described [24]. Images were digitized with Mirax Scan by Carl ZEISS (Germany). Quantification of Ki67, TUNEL and Iba-1 staining was performed in the Molecular Cytology Core Facility using whole thyroid digitized images and Metamorph software. The color threshold tool in Metamorph was used to identify DAB signal and these thresholds were applied in an automated-batch manner to all samples for each antibody. A % positive area was calculated using an Excel spreadsheet and plotted using GraphPad Prism 5.0.

### Quantitative real-time PCR

RNA was extracted from flash frozen thyroids and sorted TAMs using RNA PrepEase kit (USB Corporation, Cleveland, OH) and cDNA was generated using SuperScriptIII and random hexamers (Invitrogen). Quantitative-PCR was done using the primers listed in Supplementary Table 1.

### Treatment of mice with GW2580, a c-FMS/CSF1-R kinase inhibitor

*Tg-rtTA/tetO-Braf* mice between 6–8 weeks of age were treated with dox in the water at 5 mg ml −1 with 5% glucose for 7 days concomitantly with either vehicle or GW2580 (PLX6134) impregnated chow (provided by Plexxikon, Inc). Thyroids were harvested on day 7 for IHC as described above.

### Statistical Analyses

All statistical analyses were performed using GraphPad Prism version 5.00 for Windows, GraphPad Software, San Diego California USA, www.graphpad.com. Statistical analyses were generated using unpaired two tailed t-tests of mean and standard deviations.

## Results

### Conditional activation of BRAF in thyroids of adult mice induces PTCs that are densely populated with TAMs and CAFs

**Figure 1:**
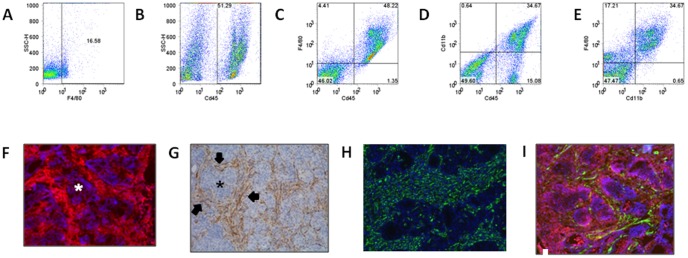
TAMs and CAFs densely infiltrate thyroid tissue during BRAF-induced PTC initiation. FACS analysis of thyroid tissue from T*g-rtTA/tetO-BRAF^V600E^* mice, in the absence (A) or presence of dox (B–E). A, Wild type thyroids have a low abundance of resident tissue Mø as measured by anti-F4/80. Seven days after dox, ∼50% of cells are Cd45^+^ leukocytes (B), that are F4/80+ and Cd11b+ (C–D). Most TAMs co-express F4/80 and Cd11b (E). FACS analyses were done on pooled wild type (no dox: n = 6) and dox-induced (n = 4) thyroids. F) IHC stain of representative frozen section of dox-induced thyroid tissue with anti-Cd68^+^ (red stain) and DAPI (blue) showing dense staining of TAMs around thyroid follicular cell nests (asterix). G) IHC with anti-αSMA+ (brown stain) demonstrates strong staining (arrows) between nests of cancer cells (asterix). H) Immunofluorescent staining with vimentin (green) is similar to that of αSMA (G, I), consistent with a dense CAF stromal compartment. I) Dual label immunofluorescence with anti-Cd11b (red) and anti-αSMA (green) did not demonstrate co-localization, consistent with the presence of two different cell types (TAMs and CAFs, respectively). F, H) Sections were co-stained with DAPI (F,H: 20× magnification; G: 10× magnification).

We used a mouse model of dox-inducible oncogenic BRAF activation in thyroid follicular cells, *Tg-rtTA/tetO-BRAF^V600E^* [24], to measure TAM recruitment and characterize TAM phenotype in the BRAF -induced PTCs. In the absence of dox, thyroid tissue has a normal histology, and there are few resident tissue Mφ, as determined by FACS with F4/80 (**Fig. 1A**). Seven days after BRAF induction with dox, mice develop PTCs that are invasive and have a high mitotic index [24]. This is accompanied by a robust recruitment of TAMs. As shown in **Fig. 1B**, approximately 50% of the cell population from PTCs of dox-induced mice is composed of Cd45^+^ leukocytes. The majority of these are F4/80^+^, Cd11b^+^ TAMs (**Fig. 1C, D, E**). The TAMs form a dense network that surrounds nests of thyroid follicular cells (**Fig. 1F, I**), as well as within the thyroid capsule (not shown). Interdigitating closely with TAMs within the tumor stroma is a dense population of CAFs (**Fig. 1G, H**). These are distinct from TAMs, as shown in **Figure 1I** by dual label immunofluorescence with antibodies to Cd11b and αSMA.

### BRAF activation results in a rapid increase in the expression of pro-tumorigenic/M2 type Mφ chemoattractants

**Figure 2:**
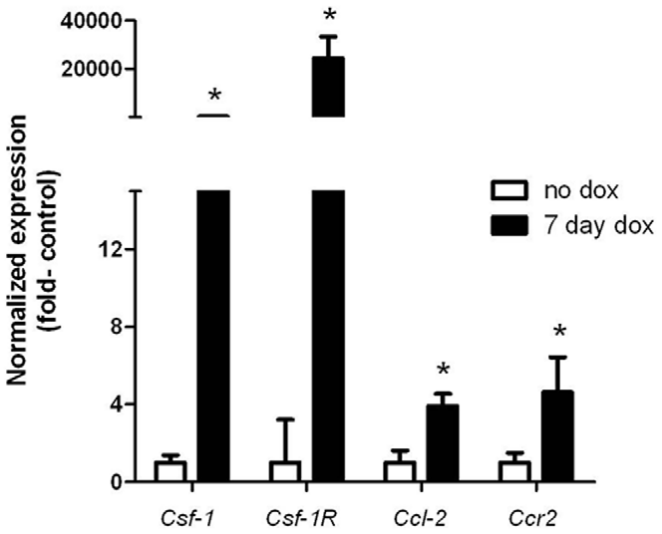
TAM chemoattractants and their receptors are overexpressed in PTCs. Quantitative RT-PCR for the indicated chemoattractants and their respective receptors in thyroid tissue extracts from T*g-rtTA/tetO-BRAF^V600E^* mice without (no dox) or 7 days after dox. Expression levels were normalized to *β-actin*. Bars represent the mean+SD from thyroids of no dox (n = 4) and 7-day dox-induced (n = 7) mice. *** p<0.001.

CSF-1/CSF-1R and CCL2 (MCP-1)/CCR2 signaling in murine breast and pancreatic neuroendocrine cancers induces TAM recruitment and a pro-tumorigenic M2-like phenotype [28], [29]. One week after dox induction in *Tg-rtTA/tetO-BRAF^V600E^* mice, there was a 250- and >20,000-fold increase in *Csf-1* and *c-fms/Csf-1R* mRNA, respectively (**Fig. 2**). The huge increase in *Csf-1R* expression likely reflects the abundant TAM recruitment, since we show that BRAF-induced murine PTC cells do not express this receptor (**Fig. S1**). Expression of *Ccl2* and its receptor *Ccr2* was also increased, but to a lesser extent (∼4-fold) (**Fig. 2**). The increased expression of the two chemokines was increased in the non-TAM cell population, composed primarily of thyroid follicular cells (**Fig. S1**). This is consistent with previous studies showing that BRAF induces expression of Mø chemoattractants in PCCL3 cells, a rat thyroid follicular cell line [30]. These data suggest that following BRAF activation, thyrocytes express CSF-1 and CCL2, which is associated with recruitment of TAMs expressing their respective receptors.

### Selective depletion of TAMs in PTCs of Tg-rtTA/tetO-BRAF^V600E^ mice impairs PTC initiation and retards stromal expansion of CAFs

In order to examine the functional role of TAMs on PTC development, we crossed *Tg-rtTA/tetO-BRAF^V600E^* with *Ccr2-DTR* mice, which express the diphtheria toxin receptor (DTR) under the control of the *Ccr2* gene promoter [31]. Bone marrow derived monocyte (BMDM) precursors require CCR2 signaling to exit the marrow [31]. Peripheral Mø do not express CCR2, except during distinct inflammatory states [32], [33]. As shown in Fig. S2A–B, the BM and blood of *Ccr2-DTR* mice treated with DT for one week had a reduction in Ly6C+ monocyte precursors (**Fig. S2A**) as well as in Cd11b^+^ and F4/80^+^ circulating monocytes (**Fig. S2**B). Resident peritoneal and splenic Mφ (**Fig. S2C, G**) also exhibited a complete and/or partial reduction in Mφ density (**Fig. S2E, H**), respectively. This was accompanied by a shift in Gr-1+ myelomonocytes precursors from the BM (Fig. S2A) and circulation (**Fig. S2B**) into peripheral sites (**Fig. S2F**).

**Figure 3:**
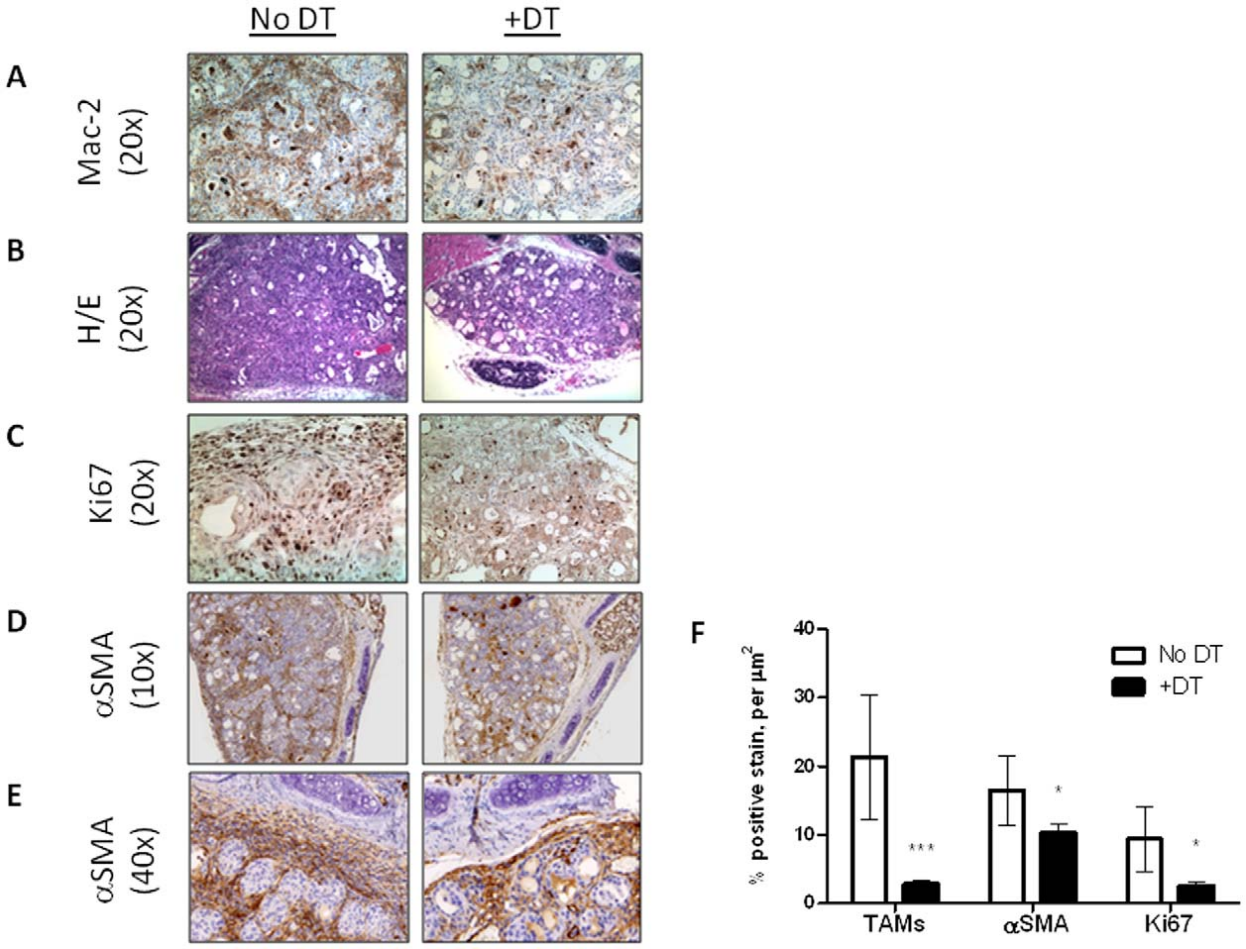
Targeting CCR2-expressing cells in BRAF-induced PTC mice results in fewer TAMs, smaller PTCs and reduced CAFs. Representative sections from T*g-rtTA/tetO-BRAF^V600E^/Ccr2-DTR* mice treated with dox for 7 days with or without intraperitoneal DT every other day: A) IHC with anti-Mac-2 shows dense brown staining within the stroma surrounding thyroid cancer follicular cells in control PTCs (*left*) that is significantly attenuated in DT-treated mice (*right*). B) PTCs from DT-treated mice are smaller and have a more preserved follicular architecture. C) IHC with Ki67 shows reduced cell proliferation, primarily within the stroma, of PTCs from DT-treated mice. D, E) IHC with αSMA showed reduced positivity in tumor capsule and intertumoral stroma. E) Higher magnification (40×) shows marked thinning of tumor capsule and depletion of αSMA positive cells in inter-tumoral spaces of DT-treated mice. F) Bar graph depicting changes in Mac-2, αSMA and Ki67 in control (n = 5) vs DT-treated (n = 6) mice. ***p<0.001, *p<0.01.

We next treated *Tg-rtTA/tetO-BRAF^V600E^/Ccr2-DTR* mice with dox for 7 days and with DT by IP injection on days 0, 3 and 6. As shown in **Figure 3A**, this resulted in a nearly complete depletion of TAMs in the tumor stroma. Histologically, this was accompanied by reduction in tumor mass with at least a partial restoration of the thyroid follicular architecture (**Fig. 3B**). Cell proliferation as measured by Ki67 staining was markedly reduced, which was most evident in the peri-tumoral and peri-follicular stromal compartments (**Fig. 3C**). Since CAFs and TAMs are the major cell types within the stroma, we hypothesize that the reduction in proliferation primarily reflects an effect on TAMs and/or CAFs. Indeed, as shown in **Figure 3D and E**, in the absence of TAMs, we observed a dramatic reduction in αSMA positive staining in the tumor capsule as well as within the thyroid between the nests of follicular cells. Treatment with DT did not affect BRAF-induced MAPK activation, as determined by IHC with phospho-ERK (**Fig. S3**). We repeated these studies using a second model of conditional TAM depletion (*Cd11b-DTR*). We observed a reduction in TAMs and αSMA^+^ CAFs following treatment of *Tg-rtTA/tetO-BRAF^V600E^/Cd11b-DTR* mice with dox for 7 days +DT, as described above (data not shown). Hence, Mφ depletion during BRAF-induced tumor initiation results in a profound remodelling of the tumor stroma, characterized by depletion of CAFs.

### PTCs of Tg-Braf mice are heavily infiltrated by inflammatory, M2 polarized TAMs

We next turned to a mouse model of established PTC, to investigate the role of TAMs in tumor maintenance and progression. The *Tg*-*Braf* mouse model closely recapitulates advanced human BRAF mutant PTCs, in that the tumor cells exhibit characteristic tall cell features [23], and progress to PDTC [23], [34]. As shown in Fig. S4, Cd68^+^ (**Fig. S4A**), F4/80^+^ (**Fig. S4B**) and Cd11b^+^ (**Fig. S4C**) TAMs surround and heavily infiltrate PTCs of 6–12 week old *T*g-*Braf* mice. To determine whether CCR2^+^ TAMs contribute to this cell population, we generated *Tg-Braf/Ccr2-GFP* mice. In wild type *Ccr2-GFP* mice, there are few CCR2-GFP^+^ resident Mφ (**Fig. S4D**), whereas in *Tg-Braf/Ccr2-GFP* animals, approximately 20% of the total cell population is composed of CCR2-GFP^+^ TAMs that co-express Cd45^+^ (**Fig. S4 E, F**).

We next determined the phenotype of TAMs obtained from established murine PTCs (8 to 15 weeks of age) by comparing the mRNA levels of M1- vs M2-related genes compared to murine BMDM (**Fig. S4G**). Enrichment of TAMs sorted from PTC single cell suspensions was confirmed by high expression of *Csf-1R*, which was comparable to that of BMDMs. Expression of the M2-related genes *Ccr2*, *arginase1*, *Ccl22 and IL-10* was higher in TAMs compared to BMDMs. By contrast the M1-specific markers *IL-12* and *ROS* were not significantly different between these cell populations (**Fig. S4G**).

### Selectively depleting TAMs during advanced stages of PTC induces tumor regression: Our data show that murine PTCs are infiltrated by M2 polarized TAMs

**Figure 4:**
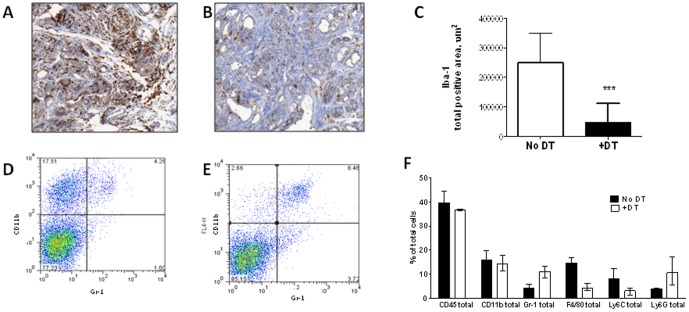
TAMs are selectively depleted in advanced PTCs of *Tg-Braf/Ccr2-DTR* mice after treatment with DT. Representative IHC with Iba-1 in PTCs of control (A) and DT-treated (B) mice. C) Quantification of Iba-1 positively staining cells from thyroid sections of control and DT-treated mice using Metamorph software. D,E) FACS analysis with anti-Cd11b and anti-Gr-1 on cell suspensions isolated from thyroids of control (D) and DT-treated mice. In the absence of DT (D) there are abundant CD11b+ Gr1- TAMs (top left quadrant). Following one week of DT (E), TAMs are profoundly depleted with a small increase in CD11b+ Gr1+ neutrophils. F) Representative analysis from FACS data for the indicated markers of TAMs and neutrophils before and after DT treatment.

**Figure 5:**
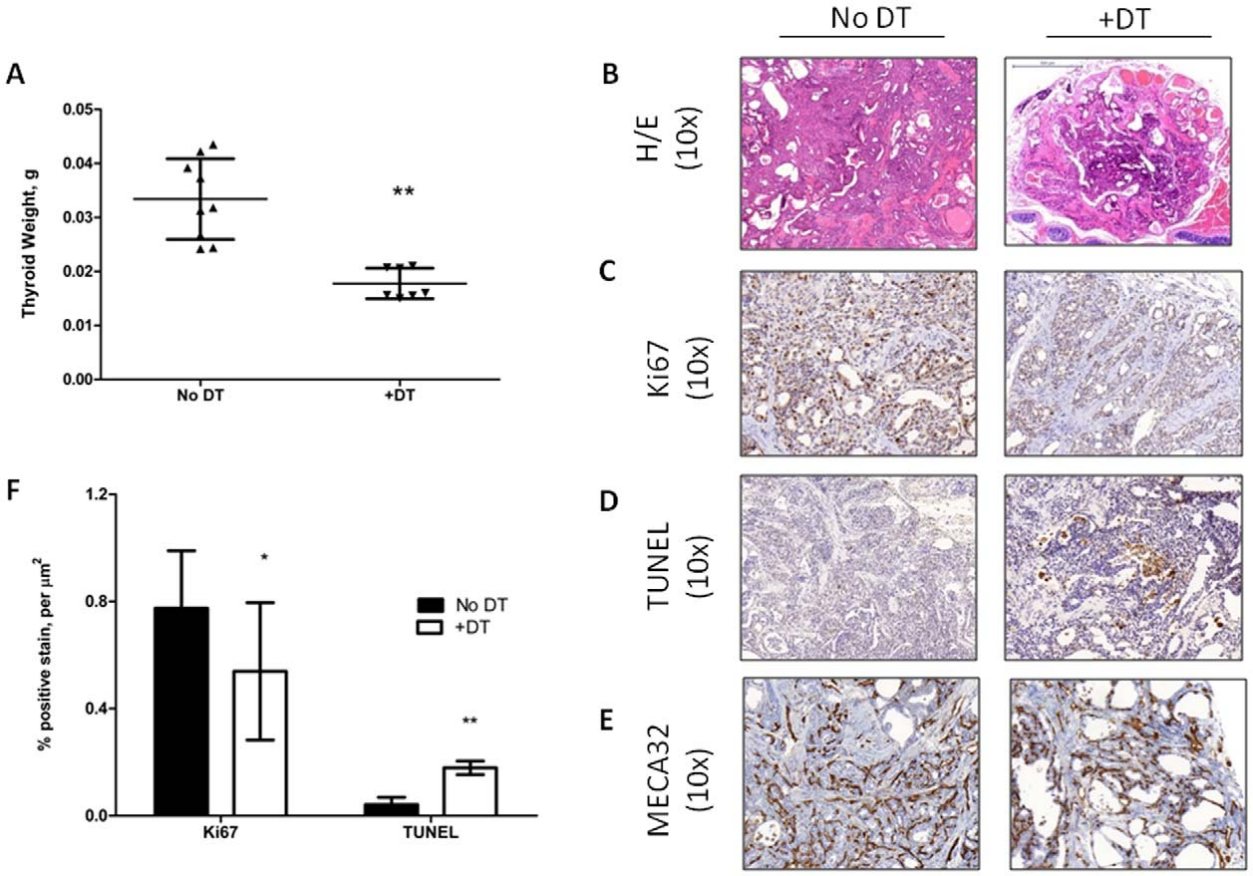
PTC regression following treatment with DT in Tg-Braf/Ccr2-DTR mice. A) Weight of thyroids of control and DT-treated *Tg-Braf/Ccr2-DTR* mice after 10 day treatment with intraperitoneal DT on alternate days. * p<0.01. B–C) Representative sections with the indicated stains. F) Quantification of Ki67 and TUNEL positive staining using Metamorph data analysis. Ki67 p<0.04; TUNEL p<0.01.

We next generated *Tg-Braf/Ccr2-DTR* mice to examine whether TAMs are required for tumor maintenance. Treatment of mice with DT for 10 days resulted in a 4–8 fold depletion of TAMs (**Fig. 4A, B, C, D, E, F**). There was a small concomitant increase of Gr1^+^, Ly6G^+^ cells, consistent with tumor-infiltrating neutrophils (TINs) (**Fig. 4D, E, F**). The TAM depletion was associated with a ∼50% reduction in tumor weight (18+/−3 vs. 33+/−7 mg in DT vs control, p = 0.0002, **Fig. 5A, B**). This was associated with a 30% decrease in Ki67 staining (0.54%/mm^2^ vs. 0.77%/mm^2^ in DT vs control; p = 0.04) (**Fig. 5C, F**), and a 4-fold increase in TUNEL+ cells (Fig. 5 D, F), primarily within areas infiltrated by TAMs. Although the total blood vessel density was lower in TAM-depleted PTCs compared to control PTCs (**Fig. 5E**), this was not statistically significant when corrected for tumor area. Advanced stage PTCs in control mice had prominent papillary structures with numerous foci of tall cells (**Fig. S5A**) and PDTC foci (**Fig. S5B**). Treatment of 6–12 week old Tg-*Braf*/*Ccr2-DTR* mice with DT for 10 days resulted in thyroid with a predominant follicular architecture consisting of colloid-containing follicles, with fewer tall cells and PDTC tumor nests (**Fig. S5C, D**).

### CSF-1/CSF-1R signaling is required for TAM recruitment and can be pharmacologically targeted to impair PTC initiation

**Figure 6:**
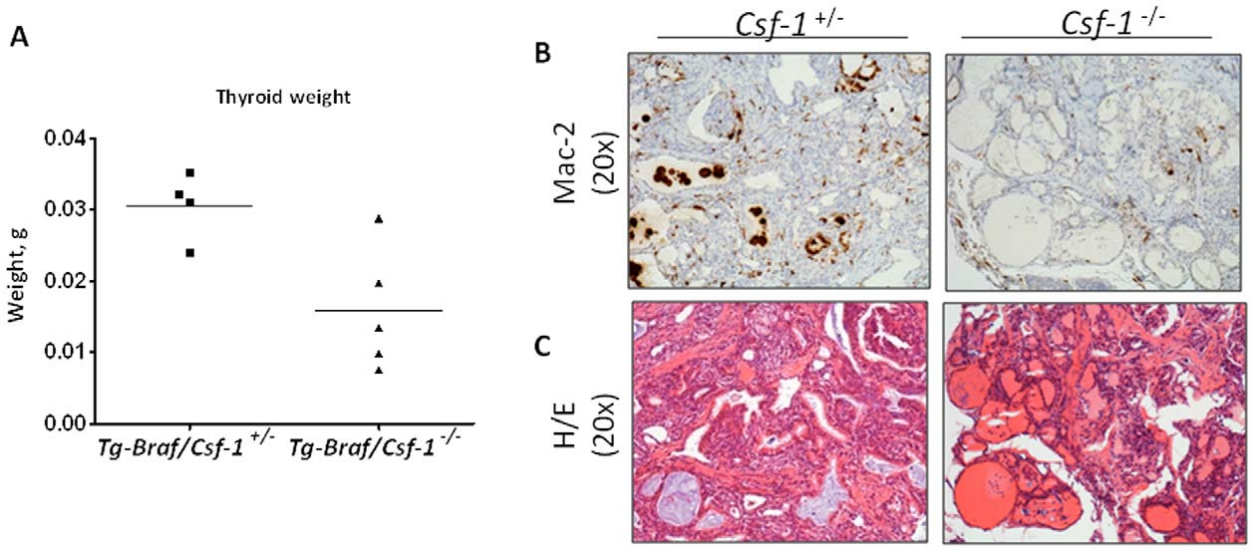
BRAF-induced PTCs derived from *Tg-Braf/Csf-1^−/−^* mice have an attenuated phenotype. A) Thyroid weight of *Tg-Braf/Csf-1^+/−^* (control) and *Tg-Braf/Csf-1^−/−^* (*Csf-1* null) mice at 6–12 weeks of age. B, C) Representative sections stained with anti-Mac-2 (B) and H/E (C) show TAM depletion and preservation of follicular architecture in PTCs of *Csf-1* null mice.

We next investigated whether CSF-1 is required for TAM recruitment by generating *Tg-Braf/Csf-1^−/−^* mice. *Csf-1^−/−^* mice have been previously described (36;37) and have normal thyroid histology. PTCs from *Tg-Braf/Csf-1^−/+^* mice are densely infiltrated with TAMs (**Fig. 6B** **, left panel**). Four out of 5 *Tg-Braf/Csf-1^−/−^* mice exhibited a significant depletion in TAMs in the PTCs (**Fig. 6B** **right panel**), which was associated with a reduction in tumor weight (**Fig. 6A**), and was accompanied by generalized preservation of the follicular architecture (**Fig. 6C** **, right panel**). TAM density was not depleted in the thyroid of one *Tg-Braf/Csf-1^−/−^* mouse, which exhibited a tumor weight (**Fig. 6A**) and histological phenotype comparable to that of control PTCs (data not shown). These data point to a significant role of CSF-1 signaling in TAM recruitment in this model, although in a minority of cases alternative signals may bypass the CSF-1 requirement for TAM recruitment.

**Figure 7:**
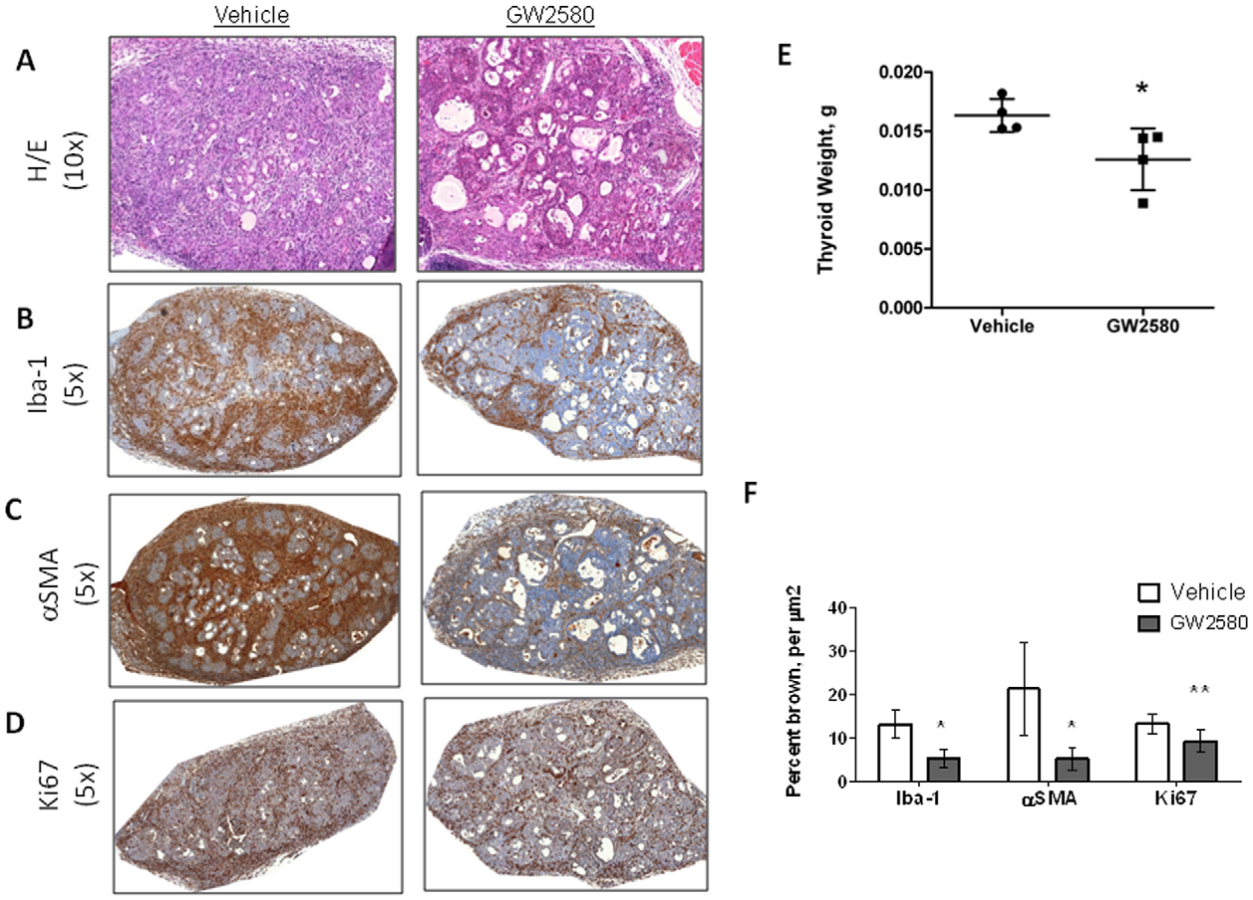
The c-FMS/CSF-1R kinase inhibitor GW2580 decreases tumor macrophages and impairs Braf-induced thyroid cancer development. A–D) Representative thyroid sections for the indicated stains from 7 day dox-induced *Tg-rtTA/tetO-Braf* mice treated with either vehicle (n = 4) or GW2580-impregnated chow (n = 4): A) H/E; B) Iba-1; C) αSMA; D)Ki67. E) Thyroid weights of dox-induced, vehicle vs. GW2580-treated mice (*p = 0.02). F) Quantification for the indicated stains; * p<0.001, ** p<0.01.

To determine whether the recruitment and/or activation of TAMs could be blocked pharmacologically, we fed dox-induced *Tg-rtTA/tetO-Braf* mice with impregnated chow containing GW2580, a c-FMS/CSF-1R tyrosine kinase inhibitor (38) for 7 days. Dox-induced GW2580-treated mice had PTCs with a ∼62% reduction in TAMs (**Fig. 7B, F**) that was associated with a significant decrease in thyroid weight (0.0163+/−0.0014 vehicle vs. 0.0126+/−0.0026 GW2580, p = 0.029; **Fig. 7A**) and a more differentiated thyroid follicular architecture (**Fig. 7A**). Stromal CAFs, as measured by αSMA, were reduced ∼77% in GW2580-treated mice compared to controls (**Fig. 7C, F**). Tumor proliferation was reduced by 31% in GW2580-treated PTCs and this reduction was most evident within the stromal compartment (**Fig. 7B, F**). These observations recapitulated the effects of genetically depleting TAMs (**Fig. 3**), strengthening the evidence that TAMs facilitate thyroid cancer progression and that TAMs are a valid target for patients with advanced disease.

## Discussion

Macrophages are highly versatile and have the capacity to differentiate into Kupffer cells, osteoclasts and/or pneumocytes in resident tissues or into inflammatory Mø subtypes in microbial infections (M1 type) or after tissue injury (M2 type). TAMs mediate a host response to the tumorigenic process, and can induce anti-tumor immunity (M1 type TAMs) or, paradoxically, enhance tumorigenesis (M2 type TAMs) [35]. Most correlative data from human cancers suggest that TAMs are protumorigenic, including in thyroid cancer [2]–[5]. In PyMT induced breast cancers, TAMs facilitate tumor angiogenesis and lung metastases but have no effect on primary tumor growth [28]. In RIP1-Tag induced pancreatic neuroendocrine tumors (PNET), CSF-1 dependent TAMs facilitate the transition from hyperplastic angiogenic islets to PNET, but have no effect on the subsequent tumor phenotype [29]. In mutant APC intestinal polyposis models, CSF-1-dependent TAMs promote polyp growth. However, their role in malignant transformation could not be evaluated in this model of benign disease [36]. Whereas these studies implicate TAMs as tumor promoters, the severely dysmorphic phenotype of the *Csf-1* knock mouse used in these studies, and the fact that this model does not allow TAMs to be depleted during defined stages of tumor development, limit the conclusions that can be derived from these experiments. Moreover, because oncogenes may induce distinct inflammatory signals which differentially impact the phenotype of TAMs, cancer models induced by oncoproteins known to be involved in disease pathogenesis may more faithfully recapitulate the function of TAMs in human cancer.

Oncogenic *BRAF*-induced thyroid cancers are ideally suited for studying the biology of TAMs in cancer progression because 1) human thyroid cancer progression to PDTCs and ATCs is accompanied by an increased infiltration of TAMs which comprise nearly ∼50% of the tumor volume in ATCs [4], [19]; 2) *BRAF* promotes progression of human WDPTCs to PDTCs and ATCs [16] and 3) activation of the BRAF-MAPK pathway in vitro in thyroid cells induces the expression of TAM chemoattractants [30]. In this study we used murine models of BRAF-induced PTCs/PDTCs to examine the role of TAMs in thyroid cancer progression. Conditional activation of BRAF in murine thyroids is associated with a significant increase in the major TAM chemoattractants, *Csf-1* and *Ccl2*. This is accompanied by a dense infiltration of TAMs and the development of PTCs/PDTCs with a short latency [24]. To examine whether there is a causal relationship between TAM infiltration and PTC development, we conditionally depleted CCR2-dependent TAMs during BRAF induction using a *Ccr2-DTR* approach. The trafficking of monocytes from the marrow into the circulation and tissues requires signaling through the CCL2/CCR2 pathway and monocytes lacking CCR2 expression become trapped in the BM [31]. Once in the circulation and/or tissues, CCR2 expression on monocytes/Mø is down-regulated during resting states but remains upregulated during inflammation [31]. The DT/*Ccr2-DTR* model is therefore a robust approach to achieve TAM depletion since TAMs are targeted directly through Mφ expression of CCR2 in tumors and/or indirectly by the depletion of BM-derived CCR2^+^ monocytes. Indeed, treatment of mice with DT during BRAF induction resulted in a nearly complete depletion of TAMs and unexpectedly, of αSMA^+^ CAFs. In mouse models, CAFs facilitate malignant transformation [37], stimulate tumor angiogenesis and remodel the extracellular matrix to enhance tumor cell invasion [38] and metastases [39]. TAMs and CAFs frequently co-localize within the stroma, yet there is little known about their interrelationship. In inflammation, increased Mφ recruitment stimulates myofibroblast expansion [40]–[42]. A similar link between TAMs and CAFs has not been established for any malignancy, and is suggested by the findings described above. This suggests that at least some of the functions of M2 type Mø within neoplastic and inflamed tissues are conserved and that non-neoplastic models of inflammation may be useful in understanding how TAMs may remodel the tumor niche.

CCR2 expression may be shared by non-myelomonocytes, including cancer cells [43], endothelial cells, [44], NK cells [45] and/or myofibroblast precursors [46]. The effects we observed are most likely mediated by CCR2-dependent TAMs because *Ccr2* expression in PTCs is restricted to Cd45^+^, Cd11b^+^ myelomonocytes and because pharmacotherapy with GW2580, a selective c-FMS/CSF-1R kinase inhibitor, phenocopied the effects of DT treatment in PTCs of CCR2-DTR mice.

Selective depletion of CCR2-dependent TAMs in established PTCs resulted in smaller tumors and decreased proliferation. We also saw fewer tall cells and decreased foci of PDTC. This is potentially significant since patients with tall cell variant PTCs and PDTC have more frequent distant metastases and a higher mortality [16]. Our results are in contrast to those observed by others using CCL2/CCR2 targeted cancer models [47]. In murine cervical cancers, the depletion of CCR2-dependent TAMs resulted in a compensatory recruitment of pro-tumorigenic tumor infiltrating neutrophils (TINs), that when depleted, impaired the tumor phenotype [47]. Although we also observed an increase in TINs in PTCs following the depletion of CCR2-dependent TAMs, these did not rescue the PTC phenotype. This was not simply due to an insufficient exposure to TINs since chronic depletion of TAMs in PTCs of *Csf-1* knockout mice also impaired the tumor phenotype. Thus, our data confirm that TAM depletion evokes a compensatory surge in TINs but at least in thyroid, this does not prevent the dampening effect on tumor phenotype.

The mouse models used in this study do not have a high enough frequency of distant metastases to allow us to explore the contribution of TAMs to the metastatic process. This is consequential, because recruitment of CCR2 positive inflammatory Mø has been shown to occur preferentially in the lung pre-metastatic niche rather than in the primary mammary tumors of late-stage PyMT-induced breast cancers, which instead recruit CCR2-negative, CSF-1 dependent TAMs [8], [28]. Besides the differences between tumor types, these data suggest that the versatility of TAM subpopulations and function may also be tumor stage specific.

Following BRAF activation, *Csf-1* is markedly overexpressed in the murine cancers. Moreover, the TAMs recruited in the early stages of tumorigenesis express high levels of *c-fms*/*Csf-1R*. Depletion of CSF-1 reduced TAM infiltration and induced smaller PTCs with a more differentiated phenotype. One *Csf-1* knock out mouse did not show significant TAM depletion in the tumor specimens. This has been previously reported in a model of mouse PNETs [29] and ascribed to potential c-FMS/CSF-1R-dependent and independent rescue pathways of TAM recruitment. Interestingly, the phenotype of PTCs in the outlier *Csf-1* knockout mice with high TAM density was similar to that of control PTCs, suggesting that compensatory signals may allow a minority of PTCs to circumvent CSF-1 and recruit pro-tumorigenic TAMs.

As TAMs promote PTC progression, these cells may be a rational therapeutic target for patients with refractory advanced PTCs, particularly PDTCs and ATCs. We show that the c-FMS/CSF-1R kinase inhibitor GW2580 phenocopied the antitumorigenic effects of genetically depleting TAMs. The PTCs from GW2580-treated mice were smaller, had fewer TAMs and CAFs and exhibited a more differentiated PTC phenotype. Our study is the first to demonstrate that targeting TAMs alone with a small molecule inhibitor impairs primary tumor progression for any cancer type. By contrast, in breast cancer [48] and glioblastoma [49] mouse models, PLX3397, another C-FMS/CSF-1R kinase inhibitor, did not affect primary tumor phenotype, but did improve the efficacy of cytotoxic chemotherapies and decreased tumor invasion, respectively. The data in thyroid models is potentially significant, particularly in view of the remarkable extent of TAM infiltration seen in patients with advanced thyroid cancers [4], [19].

## Supporting Information

Figure S1 **TAM chemoattractants and their receptors are differentially expressed in TAMs and non-TAM cell populations in PTCs:** Quantitative RT-PCR for the indicated chemoattractants and their respective receptors as well as TTF-1, a thyroid specific gene. Thyroids 6 Tg-*rtTA/tetO-BRAF^V600E^* mice treated with dox for 7 days were harvested, pooled and processed into single cell suspensions. RNA was extracted from fluorescently-labeled Cd11b positive (TAMs) versus negative (non-TAM) cell populations. Expression levels were normalized to β-actin. Bars represent the mean + SD of 3 experiments, * p<0.05.
(TIF)

Figure S2 **Ccr2-DTR mice treated with DT have depletion of bone marrow, circulating and resident monocytes/Mø.** Tissues from *Ccr2-DTR* mice treated without or with DT for one week were characterized by FACS and/or IHC to characterize the monocyte/Mø populations. A, B) Quantitative data from FACS of BM aspirates (A) and blood (B) from control and DT-treated mice using the following markers: anti-Cd11b, anti-Gr-1, anti-Ly6C, anti-Ly6G and anti-F4/80. In the BM and blood, there was a reduction in myelomonocyte precursors/myelomonocytes (Cd11b^+^, Gr-1^+^) that corresponded to a reduction in each of the monocyte (Ly6C^+^) and myelocyte (Ly6G^+^) populations. Bars represent mean +SD of 2-3 experiments. C-F) FACS analysis of a representative peritoneal lavage using anti-Cd11b, anti-F4/80 and anti-Gr-1 in the absence (C, D) or presence of DT (E, F). Peritoneal lavages of control mice are composed of resident Mø (C, Cd11b^high^/F4/80^+^) with very few neutrophils (D, Cd11b^low^/Gr-1^high^). Following DT, Mø are completely depleted (E) and replaced with neutrophils (F). G, H) Immunofluorescence of spleen from control (G) and DT-treated mice (F) with anti-Cd68 (red stain) reveals a reduction in staining within the red pulp (arrows). Magnification  = 10x, blue stain = DAPI.
(TIF)

Figure S3 **Treatment with DT does not decrease pERK staining in Braf-expressing thyroid follicular cells.** IHC for pERK in PTCs of dox-induced T*g-rtTA/tetO-BRAF^V600E^/Ccr2-DTR* mice treated without or with DT for 7 days.
(TIF)

Figure S4 **Established BRAF-induced PTCs are infiltrated with inflammatory M2-polarized TAMs.** Representative sections from PTCs of 6-12 week old *Tg-Braf* mice stained with A) anti-CD68 (red), B) anti-F4/80 (brown) and C) anti-Cd11b (green). D-F) FACS analysis from thyroids of wild type *Ccr2-GFP* (D) and *Tg-Braf/Ccr2-GFP* (E-F) mice. CCR2-GFP positive cells are increased in PTCs, and co-express Cd45 (F)), consistent with a leukocyte phenotype. G) Quantitative RT-PCR for the indicated genes from TAMs isolated from BRAF-induced PTCs of *TPO-Cre/LSL-Braf* mice compared to murine BMDM cultured in the presence of CSF-1.
(TIF)

Figure S5 **Macrophage depletion attenuates the “tall cell” phenotype and is associated with fewer poorly-differentiated foci in thyroid cancers of Tg-Braf/Ccr2-DTR mice.** A,B) Representative thyroid section of a control mouse showing a prominent area of tall cells (A, arrow) and a PDTC foci (B). The tall cell phenotype is present in nearly all PTCs of *Tg-Braf/Ccr2-DTR* mice and mostly absent following treatment with DT (C). D) Depletion of TAMs was associated with fewer foci of PDTC.
(TIF)

Table S1 **Primer sequences used in this study.**
(DOC)
