# Supplementary figures and images for: Genetic and Pharmacological Targeting of CSF-1/CSF-1R Inhibits Tumor-Associated Macrophages and Impairs BRAF-Induced Thyroid Cancer Progression
Source: PLoS One. 2013 Jan 23;8(1):e54302. doi: 10.1371/journal.pone.0054302 (PMC3553126; doi:10.1371/journal.pone.0054302)

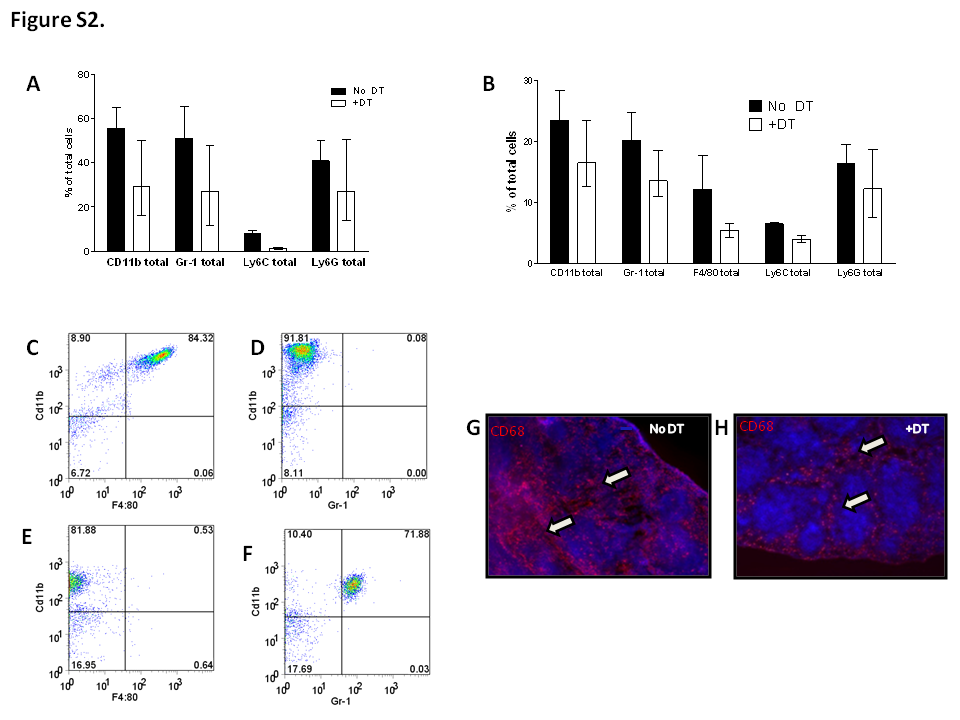

Supplement: Figure S2 — Ccr2-DTR mice treated with DT have depletion of bone marrow, circulating and resident monocytes/Mø. Tissues from Ccr2-DTR mice treated without or with DT for one week were characterized by FACS and/or IHC to characterize the monocyte/Mø populations. A, B) Quantitative data from FACS of BM aspirates (A) and blood (B) from control and DT-treated mice using the following markers: anti-Cd11b, anti-Gr-1, anti-Ly6C, anti-Ly6G and anti-F4/80. In the BM and blood, there was a reduction in myelomonocyte precursors/myelomonocytes (Cd11b+, Gr-1+) that corresponded to a reduction in each of the monocyte (Ly6C+) and myelocyte (Ly6G+) populations. Bars represent mean +SD of 2-3 experiments. C-F) FACS analysis of a representative peritoneal lavage using anti-Cd11b, anti-F4/80 and anti-Gr-1 in the absence (C, D) or presence of DT (E, F). Peritoneal lavages of control mice are composed of resident Mø (C, Cd11bhigh/F4/80+) with very few neutrophils (D, Cd11blow/Gr-1high). Following DT, Mø are completely depleted (E) and replaced with neutrophils (F). G, H) Immunofluorescence of spleen from control (G) and DT-treated mice (F) with anti-Cd68 (red stain) reveals a reduction in staining within the red pulp (arrows). Magnification = 10x, blue stain = DAPI. (TIF) [file pone.0054302.s002.tif]

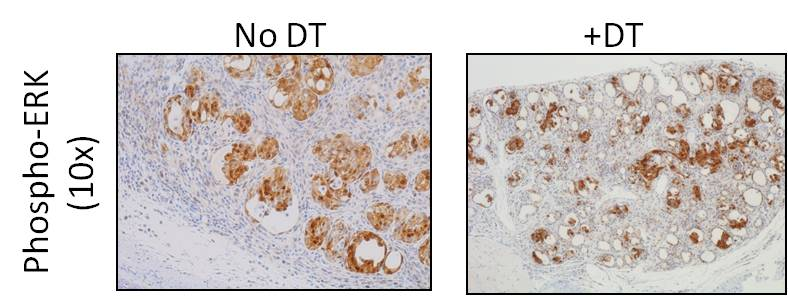

Supplement: Figure S3 — Treatment with DT does not decrease pERK staining in Braf-expressing thyroid follicular cells. IHC for pERK in PTCs of dox-induced Tg-rtTA/tetO-BRAFV600E/Ccr2-DTR mice treated without or with DT for 7 days. (TIF) [file pone.0054302.s003.tif]

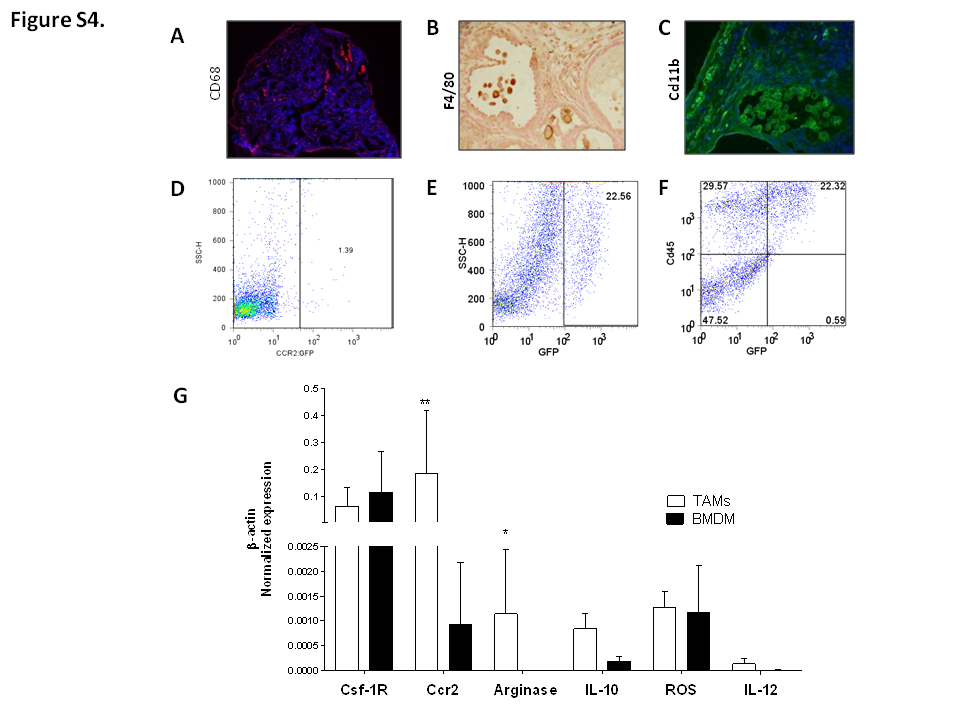

Supplement: Figure S4 — Established BRAF-induced PTCs are infiltrated with inflammatory M2-polarized TAMs. Representative sections from PTCs of 6-12 week old Tg-Braf mice stained with A) anti-CD68 (red), B) anti-F4/80 (brown) and C) anti-Cd11b (green). D-F) FACS analysis from thyroids of wild type Ccr2-GFP (D) and Tg-Braf/Ccr2-GFP (E-F) mice. CCR2-GFP positive cells are increased in PTCs, and co-express Cd45 (F)), consistent with a leukocyte phenotype. G) Quantitative RT-PCR for the indicated genes from TAMs isolated from BRAF-induced PTCs of TPO-Cre/LSL-Braf mice compared to murine BMDM cultured in the presence of CSF-1. (TIF) [file pone.0054302.s004.tif]

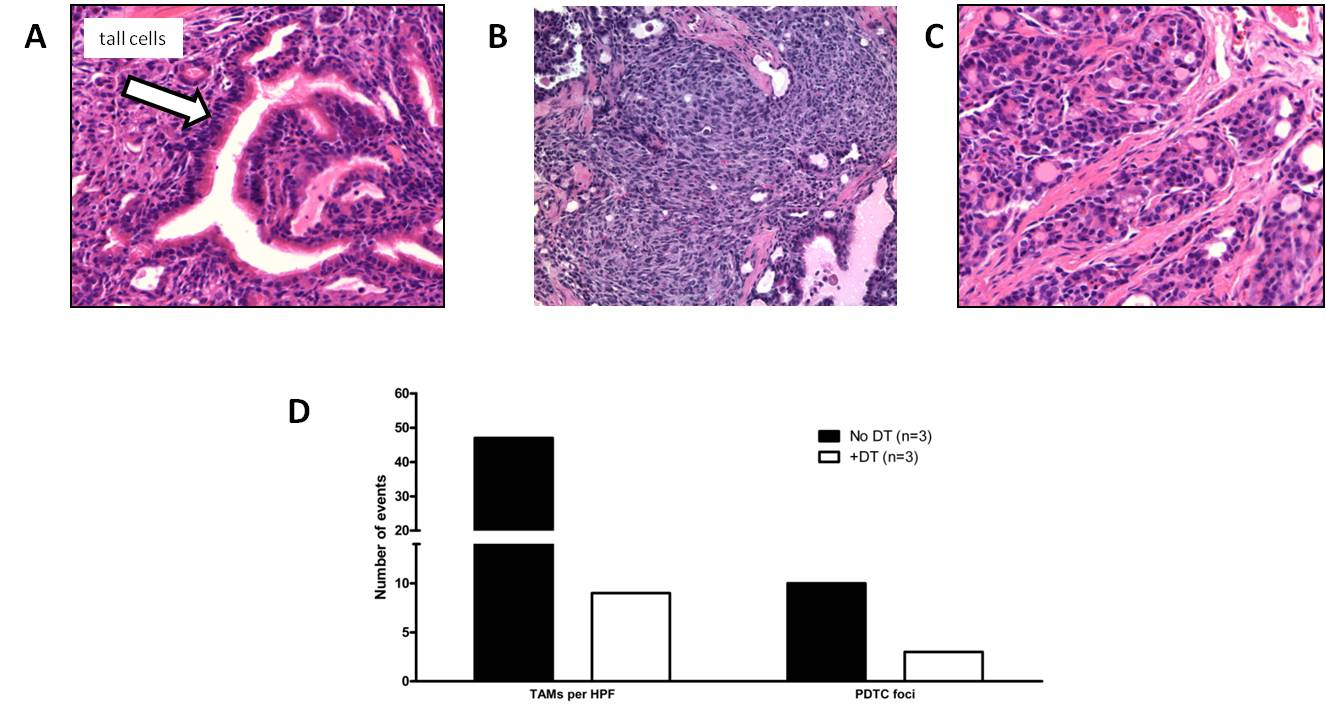

Supplement: Figure S5 — Macrophage depletion attenuates the “tall cell” phenotype and is associated with fewer poorly-differentiated foci in thyroid cancers of Tg-Braf/Ccr2-DTR mice. A,B) Representative thyroid section of a control mouse showing a prominent area of tall cells (A, arrow) and a PDTC foci (B). The tall cell phenotype is present in nearly all PTCs of Tg-Braf/Ccr2-DTR mice and mostly absent following treatment with DT (C). D) Depletion of TAMs was associated with fewer foci of PDTC. (TIF) [file pone.0054302.s005.tif]
