# Supplementary material for: Genetic and Pharmacological Targeting of CSF-1/CSF-1R Inhibits Tumor-Associated Macrophages and Impairs BRAF-Induced Thyroid Cancer Progression
Source: PLoS One. 2013 Jan 23;8(1):e54302. doi: 10.1371/journal.pone.0054302 (PMC3553126; doi:10.1371/journal.pone.0054302)
Supplement: Table S1 — Primer sequences used in this study. (DOC) [file pone.0054302.s006.doc]

**Table S1:** *Primer sequences used in study.*

| **Template** | **Species** | **Forward Primer Sequence** | **Reverse Primer Sequence** | **Annealing** |
| --- | --- | --- | --- | --- |
|  |  |  |  | **Temperature (^o^C)** |
| ß-actin | Mouse | 5'-ctgaaccctaaggccaaccgtg-3' | 5'-ggcatacagggacagcacagcc-3' | 60 |
| TTF-1 | Mouse | 5'-tccagcctatcccatctgaact-3' | 5'-caagcgcatctcacgtctca-3' | 60 |
| Csf-1 | Mouse | 5’-cagcaggtgtcccaaagaag-3’ | 5’-atttggttccgatccaggtt-3’ | 60 |
| Csf-1R | Mouse | 5-tgtgcaagaccatggtgaat-3’ | 5’-ttttatctgtgggggctctg-3’ | 60 |
| Ccl-2 | Mouse | 5’-cagcaggtgtcccaaagaag-3’ | 5’-atttggttccgatccaggtt-3’ | 60 |
| Ccr2 | Mouse | 5’-AAATGCCATGCAAGTTCAGC-3’ | 5’-ccgtggatgaactgaggtaa-3’ | 60 |
| Arginase | Mouse | 5’-agctctgggaatctgcatgggca-3’ | 5’-cagggtctacgtctcgcaagcca-3’ | 60 |
| IL-10 | Mouse | 5’-gtgaaaataagagcaaggcagtggagc-3’ | 5’-ttcatggccttgtagacaccttggtc-3’ | 60 |
| ROS | Mouse | 5’-gctgttcggcaccttctcctgtc-3’ | 5’-cgtgccgccactctgcatca-3’ | 60 |
| IL-12 | Mouse | 5’-gccagtcccgaaacctgctg-3’ | 5’-cgatgtcttcagcagtgcaggaataa-3’ | 60 |
